# Dietary Anthocyanins and Stroke: A Review of Pharmacokinetic and Pharmacodynamic Studies

**DOI:** 10.3390/nu11071479

**Published:** 2019-06-28

**Authors:** Bogdan Nicolae Manolescu, Eliza Oprea, Magdalena Mititelu, Lavinia L. Ruta, Ileana C. Farcasanu

**Affiliations:** 1 Department of Organic Chemistry “C.D. Nenitescu”, Faculty of Applied Chemistry and Science of Materials, Polytechnic University of Bucharest, 1–7 Polizu Street, 011061 Bucharest, Romania; 2 Department of Organic Chemistry, Biochemistry and Catalysis, Faculty of Chemistry, University of Bucharest, 90–92 Panduri Street, 050663 Bucharest, Romania; 3 Department of Clinical Laboratory and Food Hygiene, Faculty of Pharmacy, University of Medicine and Pharmacy “Carol Davila”, 6 Traian Vuia, 020956 Bucharest, Romania

**Keywords:** dietary anthocyanins, stroke, molecular mechanisms, blood-brain-barrier

## Abstract

Cerebrovascular accidents are currently the second major cause of death and the third leading cause of disability in the world, according to the World Health Organization (WHO), which has provided protocols for stroke prevention. Although there is a multitude of studies on the health benefits associated with anthocyanin (ACN) consumption, there is no a rigorous systematization of the data linking dietary ACN with stroke prevention. This review is intended to present data from epidemiological, in vitro, in vivo, and clinical studies dealing with the stroke related to ACN-rich diets or ACN supplements, along with possible mechanisms of action revealed by pharmacokinetic studies, including ACN passage through the blood-brain barrier (BBB).

## 1. Introduction

The anthocyanins (ACNs), along with a few other phytochemicals, are responsible for the beautiful colors of natural pigments present in foods and beverages. Fruits, vegetables or any other derived food product are often colored red, purple or blue due to ACNs or anthocyanidins (ACNDs) present in their composition. A multitude of pharmacological properties has been reported for ACNs, such as antidiabetic [1], anti-obesity [2], anti-inflammatory [3] antitumoral [4] and antimicrobial [5] activities, along with their involvement in visual health improvement [1] or in prevention and treatment of cardiovascular diseases [6]. In addition, some studies suggest a possible correlation between ACN-rich diets and a low incidence of various cardiovascular diseases (CVDs), including stroke [7,8,9].

Stroke is a major cause of death and disability worldwide, with diabetes mellitus, dyslipidemia, hypertension, and smoking among the major risk factors. A hallmark of these conditions is a redox imbalance leading to endothelial dysfunction and further to atherosclerosis. Recent in vitro and in vivo studies indicate that consumption of ACN-rich diets have a beneficial effect upon the vascular function. Thus, it appears that these compounds, by improving the endothelial function through different mechanisms, could have a protective effect against stroke.

In this review, we focused on the reports presenting evidence on the effects of ACNs in stroke, highlighting their pharmacokinetic traits, as well as their action at molecular level. Important aspects such as bioavailability, passage through the blood-brain barrier (BBB) and affinity for various transporters are also enumerated.

The passage through BBB and bioavailability of ACNs are included in the section on pharmacokinetic aspects, along with the absorption, distribution, metabolism and elimination of ACNs. In the section dealing with the evidence of ACNs’ effects on stroke in vivo, only the studies strictly targeting the effects of ACNs on stroke were included, without reference to the aspects related to neurodegenerative diseases. ACNs exert their beneficial effects at vascular endothelium level through both direct (scavenger activity against different reactive oxygen and nitrogen species) and indirect mechanisms. Apparently, the indirect mechanisms, involving the modulation of signaling pathways and transcription factors (Nrf2, NF-kB, Sp1), are more relevant in vivo. Moreover, there is a growing body of evidence that ACN metabolites—rather than ACNs—are responsible for the observed beneficial effects. Such studies and their relation to stroke prevention are also included in the sections below.

## 2. Anthocyanins (ACNs): Chemical Structures and Dietary Sources

**Figure 1:**
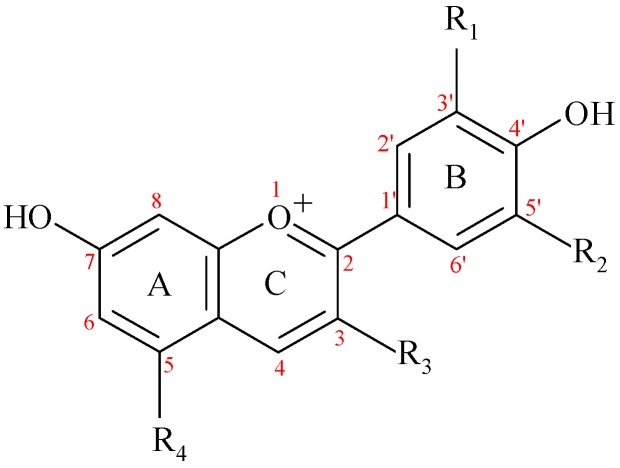
Structure of anthocyanins (R_3_ = -OH, R_4_ = -OH) and anthocyanidins (R_3_ = *β*-d-glycopyranosyl, R_4_ = -OH, anthocyanidin-3-*O*-*β*-d-glycopyranosyl; or R_3_ = *β*-d-glycopyranosyl, R_4_ = *β*-d-glycopyranosyl, anthocyanidin-3,5-*O*- di-*β*-d-glycopyranosyl) from dietary sources.

The ACNs are included in the larger class of flavonoids, along with flavonols, flavones, flavanones, flavanols, proanthocyanidins and isoflavones. They are glycosides of 2-phenyl-benzopyrylium or flavylium salts which contain a variable number of hydroxy or methoxy groups (Figure 1, Table 1). The main ACNDs (aglycones of ACNs) that occur in fruits, vegetables or wine are: pelargonidin (Pg), cyanidin (Cy), petunidin (Pt), delphinidin (De), peonidin (Pn), and malvidin (Mv). Cy is widespread in fruits and vegetables (around 50% of ACNDs), followed by De, Pg and Pn (around 12% each), while Mv and Pt are less prevalent (around 7% each) [10]. Most ACNs from foods contain one or two monosaccharide residues, such as glucosyl (glc), galactosyl (gal), arabinosyl (ara), rutinosyl (rut) or rhamnosyl (rham). The sugar moieties of ACNs are linked through the 3- or 5- positions [11].

The main ACNDs and ACNs from the human diet which are contained in the raw fruits and vegetables as well as in red wines (richest of them) are summarized in Table 1. Foods known to be rich in ACNs are berries, and significant amounts of total ACNs are found in elderberries, chokeberries, blueberries, and raspberries (black) [11,12]. Lingonberries are a type of berries belonging to the genus *Vaccinium*. A particularity of this type of berries is that fact that the level of phenolic compounds is higher in the leaves than in fruits, and this observation could be correlated with a higher radical scavenging activity. Also, it was noticed that total soluble polyphenolic content was significantly higher in lingonberry fruits when compared to blueberry fruits [13]. Quantities of ACNs from the processed food (beverages, jams, etc.) can vary greatly depending on the sources of the ACNs and their processing. Different varieties of fruits and vegetables may also contain varying amounts of ACNs.

## 3. Pharmacokinetic Properties of ACNs

The pharmacokinetic properties of ACNs (absorption, bioavailability, distribution, metabolism, elimination) have an important impact on their biological activity. Various factors can influence the pharmacokinetics of ACNs, from administration to elimination, all the more so as their interactions or biotransformations are possible even from the start, i.e., via interaction with the oral cavity [18].

### 3.1. Anthocyanin Uptake

#### 3.1.1. Oral Uptake

Studies on the oral bioavailability of ACNs are few, but they indicate that ACNs can be degraded at this level by means of several factors, such as interactions with salivary proteins, as in the case of Mv3-glc [19]. Foods or beverages intake can also influence ACN bioavailability. In this line of evidence, it was revealed that, without having other meals, the ingestion of strawberry beverages prepared with milk (but not with water) reduced the oral bioavailability of ACNs [20]. A similar reduction in anthocyanin content was noted for chokeberry anthocyanin incubated with human saliva, a loss which was attributed to the enzymatic activity of oral microbiota, high temperatures, and interactions with salivary proteins [21].

#### 3.1.2. Stomach Absorption

The evidence of gastric ACN absorption was provided by their detection in the systemic plasma and in the portal plasma within a few minutes after administration to rats [22,23]. A biologically relevant in vitro model demonstrated that ACNs actually cross the gastric mucosa barrier of MKN-28 cell monolayers (a moderately differentiated adenocarcinoma of the stomach) to gastric pH values. This experimental model, previously applied to study gastric barrier dysfunction, demonstrates the integrity of cell monolayers by evaluating transepithelial electrical resistance (TEER) as well as barrier function by immunocytochemical localization of occludin at the cell membranes [24]. These findings were confirmed by a study with bilberry extracts that were administrated to healthy subjects and ileostomists [25] and also by a study in which grape (*Vitis vinifera*) extracts were introduced directly into the stomach (proximally and distally ligated) in anesthetized rats [26]. Studies on the absorption of ACNs in the stomach revealed that they most probably cross cell membranes by some form of active transport, while passive transport is not possible due to the presence of numerous hydroxyl groups in their structure [27].

The ability of ACNs to cross the gastric mucosa was attributed to both an organic anion membrane carrier (bilitranslocase) and to glucose transporters (GLUT). In the first case, evidence showing that the transport of ACNs is facilitated by bilitranslocase is provided by the study of competitive inhibition of bilitranslocase by the quinoid forms of some ACNs from diet; also, it was found that administration of large amounts of ACNs induces bilitranslocase saturation [28,29,30]. On the other hand, studies based on the experimental model involving human gastric epithelial cells (MKN-28, moderately differentiated adenocarcinoma stomach cells) indicated that GLUT1 and GLUT3 are involved in ACN absorption and that low expression of GLUT1 and GLUT3 was accompanied by reduced of Mv3-glc absorption [31]. Using the same experimental model it was noticed that atropine (inhibitor of the organic cation transporter OCT1) and verapamil (inhibitor of a P-glycoprotein efflux transporter) also inhibit the absorption of Mv3-glc, suggesting that OCT1 may also be involved in the transport of the flavylium cation while the P-glycoprotein inhibition may increase the cytosolic concentration of the anthocyanin, resulting in a smaller quantity of Mv3-glc in the basolateral side of the gastric barrier model [31]. Other carriers have been detected in gastric mucosa (such as OAT2—organic anion transporters, SMCT1 and SMCT2—sodium/monocarboxylate transporters) that could also participate in the absorption of ACNs [29]. It is considered that ACNs are not degraded in the stomach, as they remain in the glycoside form at gastric pH (1.5–4) [23,32].

#### 3.1.3. Intestinal Absorption

ACNs are absorbed from the small intestine either by active transport using sodium-dependent glucose cotransporter (SGLT1), GLUT2 and bilitranslocase transporters [33,34,35] or by passive diffusion. The latter is only possible after the hydrolysis of ACNs to ACNDs, which are more hydrophobic [35]. The conversion takes place in the intestinal lumen and in the brush border of the intestinal epithelial cells, being catalyzed by β-glucosidase and lactase-phlorizin hydrolase [30,36]. However, the hydrolysis reaction may or may not occur, depending on the structure of the anthocyanin [37].

There are numerous studies that have explored the intestinal absorption using human epithelial colorectal adenocarcinoma (Caco-2) cell lines in order to establish the transport efficiency of ACNs and their bioavailability. These studies found very low transport efficiencies for almost all ACNs, with an average of 3% to 4% for blueberry extracts [38]. Furthermore, some ACNs from red grape skin [39] and from black currant extract [40] were not transported. On the other hand, the same experimental model revealed a high bioaccessibility for purple carrot (44.62%) and potato (71.8%) ACN [41], while pretreatment of Caco-2 cells with anthocyanin extract obtained from red grape skins (rich in Mv3-glc) showed increased expression of GLUT2 (60%) [42].

It is unanimously recognized that ACN absorption in the duodenum, jejunum, and ileum is not uniform, being dependent on the ACN structure. The duodenum has in general a low absorption rate [18,37]. The upper small intestine, especially jejunum, seems to be the main segment of bilberry ACN absorption as revealed by the amounts of these compounds recorded in plasma and urine of healthy subjects and ileostomists [25]. The study involved volunteers (with or without colon) who were administered anthocyanin-rich extracts (soluble or encapsulated in whey protein or citrus pectin). Although encapsulation did not significantly influence the bioavailability of ACNs, some modulatory effects were noticed [43]. Contradictory data were obtained in a study with similar experimental design that used homogenized raspberries which exhibited low absorption rate in the small intestine [44].

It is known that the level of ACNs’ absorption is strongly influenced by their chemical structure and by the food matrix. Thus, the rata of absorption in a study which utilized in situ perfusion of the jejunum and ileum in rats were Mv3-glc < Cy3-rut < Cy3-gal < Cy3-glc being considered a fast and efficient absorption [45]. ACNs are hydrolysed to ACNDs through removal of the 3-*O*-glycosidic moiety, due by colon microbiota, which are mainly represented by bacterial species from the genera *Bifidobacterium*, *Bacteroides*, *Eubacterium*, and *Clostridium*, with the large intestine containing in total approximately 300–500 species [30]. Apparently, the glucosyl residue is more susceptible to hydrolysis than either galactosyl or arabinosyl residues [46]. The gut microbiota has a special role regarding ACN metabolism; this was seen in antibiotic-treated mice whose faeces had increased anthocyanin contents. Apparently, De-type ACNs are less sensitive than cyanins to microbial degradation in the large intestine [47]. The gut microbiota composition is a peculiarity to each individual, therefore the variability of anthocyanin metabolism at this level is also expected to be high. This generated in vitro studies on bacterial species capable to metabolize ACNs [48,49,50], as well as studies on ACN faecal excretion under normal physiological conditions [51], but further studies are necessary to fully understand these aspects. The catabolytes obtained from Cy3-glc and Cy3-rut, under the influence of intestinal bacteria, were Cy, protocatechuic acid, protocatechuic acid glucoside, vanillic acid, 4-coumaric acid, 2,4,6-trihydroxybenzaldehyde, caffeic acid, 4-hydroxy-benzoic acid, catechol, and tartaric acid [32,52,53,54]. Mv3-glc may generate in the same segment of intestine the following catabolites: syringic acid, GA, 2,5- dihydroxyphenylacetic, 4-coumaric acid, sinapic acid, while De3-glc and De3-rut could yield: GA, 2,5-dihydroxyphenylacetic acid, 4-coumaric acid, sinapic acid, 2,4,6-trihydroxybenzaldehyde, 4-hydroxybenzoic acid, pyrogallol [32,53,55]. Pn, Pg and Mv are catabolized by gut microbiota to vanillic acid, 4-hydroxybenzoic acid and syringic acid, respectively [56].

### 3.2. Bioavailability

In pharmacology, drug bioavailability is defined as the fraction of an ingested substance that reaches the systemic circulation, being expressed as the percentage of the amount absorbed unchanged after oral administration reported to the total administrated amount, considering that intravenously administrated drug has 100% bioavailability [57]. As ACNs are not drugs, many authors refer to bioavailability and also to bioaccessibility when they deal with anthocyanin pharmacokinetics. Both could be regarded as bio-efficiency parameters, although bioaccessibility is more specific for the digestion and absorption efficiency [32]. Bioaccessibility is defined as the percentage of the compound that is released in the gastrointestinal tract reported to its content in the initial matrix, thus becoming available for absorption and it is evaluated in general, by in vitro methods of digestion simulation (in stomach or gut levels) [58].

Several reports have indicated that the bioavailability of ACNs is approximately 1% and sometimes even lower, taking into account that only the parent compounds and/or main metabolites such as phenolic acids are considered. Some structural and pharmacokinetic factors may contribute to this low bioavailability: the high polarity of these molecules and consequently low absorption rate, the first pass metabolism, restricted stability during passage through the gastrointestinal tract caused by variation of pH or by the presence of microflora [43]. Nevertheless, recent studies claim that the overall bioavailability could be higher than previously estimated if taking into account conjugated products, unmetabolized parent compounds, metabolites resulting from xenobiotic and bacterial metabolism [18,31,59,60]. Moreover, in a study using isotope-labelled Cy3-glc, the relative bioavailability of ^13^C-Cy3-glc was established as 12.38 ± 1.38%, calculated from the combined elimination through urine and breath [61].

It was noticed that the presence of some compounds or nutrients in the food matrix [62] along with ACNs may increase their bioavailability, e.g., phytic acid (from seeds, grain and hulls of nuts) [63] or cream [35] by slowing gastrointestinal mobility and prolonging their contact time.

### 3.3. Distribution

To establish the distribution, accumulation and/or elimination of ACNs, studies on blueberry-fed pigs [64], on blackcurrant [65] and on bilberry-fed rats [66] provided evidence that ACNs are present in plasma [66], liver [64], eye [64,65] and brain [64]. In a separate study, after oral administration of Pg to rats, neither Pg nor its metabolites were detected in the spleen and heart, but they were detected in brain and lung (as Pg and Pg glucuronide) [67].

#### 3.3.1. Anthocyanins in Blood Circulation

There are many studies on various ACNs administrated to humans from various sources: fruits and vegetables (fresh or in the form of powder), extracts, beverages (juices, wines, concentrated drinks). These studies present parameters such as maximal plasma concentrations (Cmax) of ACN, or time to reach Cmax [30]. Other studies monitored the administration without taking into account the quantity of anthocyanin in accordance to body weight, leading to significant differences between results. Moreover, the food matrix the presence of other flavonoids or of phenolic acids may also induce significant differences [45,60]. Other studies shed light on the provenience of the metabolites or degradation products found in the serum (also in urine, faeces, breath), as deriving from the A- or B-rings of anthocyanin (Figure 1) [61,68]. Using orally administrated isotope-labelled Cy3-glc isotopic-labeled, 17 compounds with ^13^C were found in the serum, with hippuric acid and ferulic acid as the most important phenolic metabolites [68].

#### 3.3.2. Interactions between ACN and Blood-brain Barrier (BBB)

The endothelium capillaries with tight junctions and lack of fenestrations represent a BBB which protects the central nervous system (CNS) from xenobiotic/toxic compounds and pathogens. At the same time, BBB is a dynamic interface that delivers essential compounds (nutrients, hormones and drugs), and removes metabolites from the brain. The percytes, astrocytic endfeet, microglia and neurons were also associated to this complex interface [36,69]. According to literature data, anthocyanins are able to cross the BBB (their metabolites also, in some cases) and exert neuropharmacological actions at the molecular level, influencing signaling pathways, genetic expression and protein function (see mechanism of action). Cell culture models that mimic BBB for in vitro drug transport studies in the brain and for studies targeting endothelial cell biology and pathophysiology have been developed. [70]. The evolution of these models, performed on primary cells or immortalized endothelial cell lines of the brain, were partly applied in the interaction studies between BBB and anthocyanins.

ACN uptake by brain cells was tested using mouse cerebral capillary endothelium cells (b.END5) and rat cerebral capillary endothelium cells (RBE4) cell lines, revealing that Cy3-rut, Pg3-glc, and to a lesser extent hesperetin or naringenin (other flavonoids) could cross the BBB [71]. Both bEND5 and RBE4 are immortalized cell lines, commercially available, well-characterized, but with low TEER (around 50 Ohm cm^2^ for b.END5 and similar for RBE4). RBE4 cells have been considered to mimic part of the BBB characteristics such as expression of P-glycoprotein, high alkaline phosphatase and gamma-glutamyl transpeptidase activity [72] while the expression of endothelial cell specific proteins (vascular endothelial-cadherin (VE-cadherin), von Willebrand factor, platelet endothelial cell adhesion molecule-1, endoglin, intercel-lular adhesion molecule 2 (ICAM-2), and claudin-5) are well-known for bEnd5.

It was found that Cy3-glc has a high ability to cross RBE4 cells (only cell monolayers with TEER > 100 Ohm cm^2^ were considered), and the uptake decreased in the presence of ethanol, a trait that could be important for the wine ACN [73]. A better alternative of these are mouse cerebral and cerebellar capillaries cell lines, cEND (with TEER between 300 and 800 Ohm cm^2^) and cerebEND, that form monolayers and have higher occludin and claudin-5 expression at the tight junctions, but from our knowledge, these cell lines have not used in ACN studies [70,74].

Human brain endothelial cell (hCMEC/D3) monolayers express the characteristic tight junction proteins of the BBB in spite of a low expression level of claudin-5 by comparing to intact microvessels, while TEER values is varying between 30 and 50 Ohm cm^2^ (which explains the permeability for urea, sucrose, mannitol, sodium fluorescein, and Lucifer yellow and lower permeability for 4 kDa dextrans). The use of this cell lines has allowed the investigation of endothelial cell transporters of the human brain, signaling pathways, receptors, and metabolism and, in addition, some parameters of the monolayers formed by the hCMEC/D3 cells could be enhanced by optimizing culture conditions [70]. Dp3-glc, Cy3-glc and Mv3-glc were shown to cross hCMEC/D3 cell monolayers (were only considered cell monolayers that maintained TEER > 100 Ohm cm^2^), in a manner dependent on incubation time and hydrophobicity index [69].

ECV304 cells co-cultured with C6 glioma cells (a human cell line expressing an endothelial phenotype and rat glioma cells), with an enhancing tight junctional organization and increased TEER, were also used in order to study the interaction of the blood–brain barrier and ACNs (Table 2) [71].

The overall low yield of endothelial cells obtained from the rat and mouse brains represent a limitation of the widespread use of murine endothelial cell models [70]. In addition, neurovascular unit (NVU) is currently targeted to study neuronal destruction and protection mechanisms and involves neurons and BBB, including vascular endothelial cells, glial cells (astrocytes, microglia) and extracellular matrix (ECM) [75].

A new experimental model that includes three cell types: endothelial cells seeded on the upper surface of a support, pericytes seeded on the lower surface, and astrocytes on the bottom of the culture wells was recently developed. Although this 3D model mimics very well NVU regarding interaction and signaling between cells, it is little widespread so far. This model, feasible for preclinical studies, solves the yield issue but it is more laborious, requires experimental skills and a more workload [70]. In recent years, significant efforts have been made to develop experimental models that could integrate all elements of the NVU, and which, in some cases, allow the identification of the contributions of each cell type at the same time. Thus, Maoz et al. has modeled the human NVU in order to provide new information about effects, toxicity, transport and mechanism of methamphetamine, a psychoactive drug, using microfluidic organ chips [76].

The use of pluripotent stem cells (iPSC)-derived brain endothelial cells represents a reliable solution that could be applied to study antibody transcytosis across the BBB. iPSC-derived brain endothelial cells function in vivo as a barrier, seem to be compatible with other cells (astrocytes and pericytes), and express relevant junctional proteins and transporters. Wevers et al. established a perfused BBB on-a-chip model that accomplished high-throughput readouts in physiologically conditions, without artificial membranes, useful for drugs screening, including large molecules as antibodies [77] and more recently the first BBB chip based on stem cells has developed [78].

The most important ECM-degrading enzymes in vivo that are related to the destruction of the BBB are matrix metalloproteinases MMP-2 and MMP-9 [79]. They play a key role in interrupting BBB after stroke, and studies have shown that MMP-9 is overexpressed in brains suffering from cerebral ischemic lesions and that they promote brain damage and BBB failure [80]. In vivo experiments performed on rat induced stroke suggested that Mv [81] and petunidin-3-*O*-rutinoside (*p*-coumaroyl) -5-*O*-glucoside [82] can be considered inhibitors of MMP-9 activity.

While some drugs were conjugated with glucose to enhance their access into the brain by targeting the GLUT1 transporter (e.g., nonsteroidal anti-inflammatory drugs) [83], it can be speculated that ACNs get into the brain due to their sugar moiety using a GLUT1 transporter [73], a hypothesis supported by the GLUT-dependent active transport of ACNs in the stomach [31] and small intestine [31,34]. Transport of Cy3-glc and Mv3-glc in human endothelial cells by bilitranslocase was previously demonstrated, representing another possibility for their transport into the brain [27].

It is known that multi-drug resistance protein 1 (MDR1, P-glycoprotein) and the breast cancer resistance protein (BCRP) are ABC efflux transporters and both are expressed at BBB level (as well as in small intestine, placenta, liver, etc.). It was established that ACNs and ACNDs have various affinities for the human efflux transporter BCRP. Thus, Cy and Pn seem to be potent inhibitors of BCRP, while Cy3-rut, De3-glc, Mv3-glc, Pg3,5-diglc, Mv3-gal have a lower inhibitory capacity. Modest affinities of ACNs and ACNDs for MDR1 were also recorded [84].

Studies aiming for the identification and quantification of ACNs (and sometimes their metabolites) reaching the CNS usually aim to establish the bioavailability of ACNs, and sometimes their pharmacokinetics (absorption, distribution or elimination). These studies concerning ACN administration in various forms (extracts, pure substances, etc.), either in a single dose or over a longer period of time, are summarized in Table 3. The amount of ACN reaching the brain level is proportional to the amount administered [64] but the amount reaching the brain tissue it is not necessarily proportional to the serum concentrations of ACN [85]. It can also be estimated that the distribution of ACNs in different regions of the brain is uneven, although there are a few studies investigating this aspect: in cerebellum, cortex, hippocampus or striatum of BBS rats [86]; in cortex and cerebellum, in pig [64]; in cerebellum, brain stem, medial frontal cortex, hippocampus, hypothalamus, and amygdala of piglets [87]; in cortex, cerebellum, and midbrain and diencephalon, in pig [46]. In piglets, a dose of 82.5 mg bilberry extract/kg of body weight (bw) led to higher concentration of ACN (Mv3-gal, Mv3-glc, Pt3-gal, Pt3-glc, Pn3-gal, Pn3-glc, Cy3-gal, and Cy3-glc) in cerebellum when compared with other brain regions [87] (Table 3). Mv3-glc was the main compound in all analyzed samples from brain tissues [46]. It is possible that a part of it comes from the diet and the other part could be result of the C*O*MT (catechol-*O*-methyl transferase) action on other anthocyanin, e.g., Pt3-glc [88]. This transformation could occur in liver, as well as locally, in brain, and may also be valid for conversion of Pn3-glc to Cy3-glc. This would explain the presence of Pn3-glc in brain tissue even if it lacked in blueberry feeding [46,64].

Glucosides of ACNs tend to be present in larger amounts compared with arabinosides and galactosides, either in the cortex or in the cerebellum, with slightly higher values in the cerebellum [64].

Intravenous administration of Cy3-glc and a mixture of polyphenol microbial metabolites (inclusive of the ACN metabolites) in anaesthetized rats indicated that GA [94] and Cy3-glc [93] appear in brain very fast, only 15 s after the administration. It was shown that 13 phenolic compounds that are normally present in the brain tissue—mainly the catabolites of catecholamine, dopamine or other endogenous compounds—could also be metabolites of some ACNs, e.g., vanillic acid [94]. Some phenolic compounds have a biphasic absorption into the brain tissue: caffeic acid, 4-hydroxybenzoic acid, gallic acid [94]. After intravenous administration [93], the decrease of Cy3-glc in the brain parallels the decrease in plasma, which means that Cy3-glc does not accumulate in the cerebral tissue. No high interindividual variability in BBB permeability was observed, and blood Cy3-glc concentrations are in balance with those in the brain tissue. Therefore, the plasma concentrations of Cy3-glc could give information about its levels in the brain. Other studies are less encouraging, reporting that no ACNs were detected in brain tissue of mice either on a diet containing 0.5% of bilberry extracts, for 2 weeks [95] or with a wild blueberry-supplemented diet (8%) for 4 or 8 weeks [96].

### 3.4. Metabolism

The metabolism of ACNs begins within the enterocytes as with other xenobiotic compounds; it comprises phase I and II reactions, and continues with microbiota-mediated anthocyanin metabolism [18]. The main types of reactions are hydroxylation (phase I reaction, in the presence of cytochrome P450 isoforms) and conjugation reactions (phase II reactions) which occur in the small intestine and liver. The ACND become glucuronide, sulphate or methyl derivatives, through phase II reactions, while ACN seem to be mostly deglycosylated as evidenced by LC-MS/MS in human urine after intake of blueberry juice, when the aglycone metabolites were 91% of the total metabolites of ACNs and their aglycones [60]. In addition, two other enzymes present in the human body are also involved in the biotransformation of ACNs: (1) the enzyme uridine 5′-diphosphoglucuronosyl transferase (UDP-glucuronosyltransferases or UGTs), constitutively expressed in small intestine, colon, and liver, plays an important role in glucuronidation reactions [60], although it cannot react easily with flavonoid glycosides, possible due to polarity and size of these molecules [97]; and (2) the COMT enzyme, localized in the gut wall (as well as postsynaptically), responsible for methylation reactions [57]. The xenobiotic reactions, as well as bacterial action on ACNDs, through possible dehydroxylation and demethylation reactions, could explain the prevalence of anthocyanin metabolites derived of Pg (absent in blueberry juice) in humans’ urine after juice blueberry administration [60]. The enterohepatic recycling is supported by the presence of a significant amount of ACN metabolites in urine after 5 days [60] or even after one week [98] following the last administration of ACNs, confirmed their enterohepatic recycling.

There is no doubt about the complexity of the resulting compounds in vivo following the metabolism of ACNs. It is believed that it is possible to form any aglycone under the conditions in which methylation and hydroxylation reactions of the isolated phenyl residue in their structure can be successively carried out by phase I and II reactions to which they are repeatedly subjected (following the enterohepatic circuit). In addition to these, there is also the possibility of forming chalcones at physiological pH through the opening of the C-ring of ACDN hemiketal form resulted from slow hydration of the flavylium cation; it was shown that chalcones are intermediates in the formation of phenolic acids after the administration of ACNs [60].

### 3.5. Elimination

ACN metabolites are removed from the body in urine, bile, feces and breath [30,61,68]. Urine is the primary route of ACN elimination during the first 6 h, while faeces are the predominant elimination pathway over the 6–24 h and 24–48 h time intervals [61]. The parent ACN appeared in human urine in low amount (4%) after blueberry juice ingestion [60] or 5.37% after Cy3-glc administration [61]. However, among polyphenols, ACNs have the lowest urinary recovery after oral administration and they occur in urine most often in the form of glycosides [99]. Renal absorption and possible renal elimination mechanisms of the ACNs were investigated [26,99] revealing that most likely the ACNs are eliminated at this level by glomerular filtration and tubular secretion. A bilitranslocase isoform is present in the basolateral domain of the tubular cell membrane [100], being involved in the elimination of ACNs by tubular secretion [26]. COMT also plays an active role by metabolizing ACNs and targeting them in the tubular lumen.

## 4. Molecular Mechanisms of Action

ACNs act at a molecular level through complex mechanisms involving either a direct limitation of oxidative stress, or modulation of several signaling pathways leading to alteration in genes’ expression. Recent studies revealed that these compounds can also act by altering miRNA expression [101,102]. It is well documented that a major site for ACN action is the vascular endothelium, which explains their ability to improve vascular tonus.

Reactive oxygen and nitrogen species (ROS/RNS) are byproducts of several reactions which take place inside cells in physiological conditions [103,104]. This oxidant challenge is termed oxidative eustress and it is fundamental for the redox regulation of many physiological processes (vascular tone, control of ventilation, erythropoietin production, cellular growth, prostaglandin biosynthesis, signal transduction, etc.) [105]. The loss of this balance leads to oxidative stress which is characterized by “an imbalance between oxidants and antioxidants in favor of the oxidants, leading to a disruption of the redox signaling and control and/or molecular damage” [106]. Oxidative stress is involved in the pathogenesis of many clinical conditions, such as acute and chronic kidney disease, neurodegenerative diseases, cancer, cardiovascular disease, including stroke, diabetes mellitus, hypertension, dyslipidemia, etc. [107]. There are several conditions that are characterized by a redox imbalance which is responsible, at least in part, for endothelial dysfunction leading to atherosclerosis [108,109]. Endothelial dysfunction is a term used to describe a state characterized by a pro-inflammatory and pro-thrombotic phenotype of the endothelial cells, reduced NO^●^ bioavailability and impairment of the vascular tone [110].

Several recent meta-analysis and systematic reviews indicated a clear health benefit brought by ACN consumption (as berry extracts or purified compounds). One meta-analysis of 22 randomized-controlled trials indicated that consumption of ACN-rich berries (2–12 weeks) significantly lowered body mass index (BMI) (*p* < 0.00001), LDL cholesterol (*p* = 0.003), fasting blood glucose (*p* = 0.004), HbA1c (*p* = 0.04) and TNF-α level (*p* = 0.04) [111]. A systematic review of 12 randomized-controlled trials indicated that the consumption of either ACN-rich extracts or purified ACN improved blood pressure and LDL cholesterol in post-myocardial infarction and dyslipidemia [112]. A more recent meta-analysis focused on the relationship between the consumption of ACNs and ACN-rich foods and extracts and the vascular function [113]. The authors used a total of 29 studies consisting of both acute and chronic (short- and long-term) interventions (days, weeks, months). These studies included both healthy and diseased subjects. ACNs were used as fruit extracts, except for two studies which used purified compounds. This meta-analysis indicate that ACN consumption was associated with a significantly improvement of the vascular endothelial function evaluated through flow-mediated dilation (FMD) following acute (SMD: 3.92%, 95% CI: 1.47, 6.38, *p* = 0.002; *I*^2^ = 91.8%) and chronic supplementation (SMD: 0.84%, 95% CI: 0.55, 1.12, *p* = 0.000; *I*^2^ = 62.5%). Also, acute supplementation was associated with pulse wave velocity improvement (SMD: −1.27 m/s, 95% CI: −1.96, −0.58, *p* = 0.000; *I*^2^ = 17.8%). Taken together, these studies clearly demonstrated the beneficial health effects associated with the consumption of ACN and indicate vascular endothelium as a major target for their actions.

ACNs exert their bioactive properties through direct and indirect mechanisms. Thus, these compounds can act as direct antioxidants by suppressing ROS formation in different experimental settings. On the other hand, it seems that the indirect mechanisms, involving the modulation of cellular signaling pathways and the activity of some transcription factors (Nrf2, NF-kB, Sp1) could be more relevant in vivo. Moreover, recent studies indicated that ACNs’ metabolites, rather than ACN, are responsible for the observed beneficial effects [114,115,116,117,118,119,120]. Also, it is important to underline the fact that only some studies used ACNs or their metabolites at physiologically relevant concentrations [115,116,118,119].

ACNs are classified as direct antioxidants because of their ability to scavenge different ROS/RNS, like the O_2_^●−^ and peroxynitrite anions, and HO^●^ and NO^●^ radical species [121,122,123]. This ability was investigated in relation to the structural peculiarities of these compounds. Thus, it was found that the O_2_^●−^-scavenging ability of the ACNs having three hydroxyl groups on the ring B decreases with the increasing degree of *O*-methylation degree: De > Pt > Mv [122]. A similar situation was found in the case of ACNs having two hydroxyl groups on the ring B, as C had a higher scavenging activity than Pn. When comparing the two groups, it was found that the O_2_^●−^-scavenging activity decreases as follows: De > Pt > Mv ≈ Cy > Pn > Pg, with Pg having the lowest scavenging activity. Blocking the hydroxyl group bound in the 4′ position of the ring B led to a significant loss of the scavenging activity as 4′-*O*-methyl-delphinidin 3-*O-β*-d-glucopyranoside, the major metabolite of De3-glc, had the lowest activity. This finding suggested that the 4′-hydroxyl group is fundamental for the O_2_^●−^-scavenging activity. Evaluation of the relationship between the scavenging activity and the sugars attached to the aglycone found that glucopyranoside had the highest activity, followed by galactopyranoside and arabinopyranoside [122]. The same study found that the peroxynitrite anion-scavenging activity decreased as follows: De > Cy ≈ Pt > Mv > Pn > Pg. Once again, the 4′-hydroxyl group was proved to be fundamental for the peroxynitrite anion-scavenging activity. This activity was not influenced by the sugar moieties. Moreover, the authors found that there is a synergism between these compounds when used as mixtures. It is important to keep in mind this result, as many studies concerning ACNs use different types of extracts. A different study evaluated through ESR spectroscopy the free radical scavenging activities of De, Pg, and Mv towards the HO^●^ and NO^●^ radicals [123]. It was found that all three compounds displayed selectivity for the HO^●^ radical, with De and Pg being more reactive than Mv. The authors proposed that (i) an intramolecular hydrogen bonding involving the hydroxyl group bound in the 3 position, and (ii) a reduced conjugation in Mv due to the loss of ring B coplanarity in respect to the rest of the molecules are factors responsible for the lowered antiradical activity of Mv. The antioxidant activity of ACNs was also evaluated by their ability to inhibit the oxidative modifications of LDL particles in different condition [124,125].

ACNs produce some of their biological effects through the activation of the Nrf2-Keap1-ARE stress-response pathway [126]. Exposure to electrophilic xenobiotics or oxidative stress modifies some critical cysteine residues in Keap1 leading to a buildup of Nrf2 in the cytoplasm, followed by nuclear translocation. As a consequence, there is an enhancement of gene transcription for genes coding for cytoprotective proteins: glutamate cysteine ligase catalytic (GCLC) and regulatory (GCLM) subunits, γ-glutamylcysteine synthase (GCS), glutathione S-transferase (GST), glutatione peroxidase (GPx), glucose-6-phosphate dehydrogenase (G6PDH), sulfiredoxin 1 (SRXN1), thioredoxin reductase 1 (TXNRD1), heme oxygenase 1 (HO-1), NADPH quinone oxidoreductase 1 (NQO1), UDP-glucuronosyl transferases, etc. [127]. Thus, activation of the Nrf2-Keap1-ARE pathway leads to increase synthesis of reduced glutathione (GSH) and inactivation of the electrophilic xenobiotics and ROS/RNS.

ACNs are able to modulate intracellular levels of different enzymes involved in ROS metabolism. For example, Mv and two of its glycosides (Mv3-glc, Mv3-gal) decrease the level of xanthine oxidase (XO), while increasing the levels of the cytoprotective antioxidant enzymes SOD and HO-1 in endothelial cells (HUVECs) [111]. The same study showed that Mv3-glc and Mv3-gal where better cytoprotection inducers than Mv, Mv3-glc being more effective than Mv3-gal. Another study indicated that pretreatment of pancreatic β cells with an extract rich in ACN induced HO-1 gene expression offering protection against H_2_O_2_ oxidative injury [128]. It was also found that physiologically relevant concentrations (100 nM–1 μM) of De and its metabolite, gallic acid, induced an increase in intracellular glutathione level [117]. As already mentioned, ACN’ metabolites, rather than ACN, are responsible for the observed beneficial effects. Thus, pretreatment of human umbilical vein endothelial cells (HUVECs) with ferulic acid (0.1–10 μM) upregulated the genes coding for GCLC, GCLM, NQO1, and HO-1, leading to an increase in intracellular glutathione and NADPH concentrations and providing protection against radiation induced oxidative stress [114]. Recently, it was found that the pretreatment (18 h) of HUVECs with mixtures of blueberry-derived phenolic acids (protocatechuic, 2-hydroxyhippuric, 4-hydroxyhippuric, syringic, gentisic, vanillic, *trans*-ferulic, *p*-coumaric, dihydroferulic, dihydrocaffeic, dihydro-*m*-coumaric, and homovanillic acids) that mimic plasma polyphenol metabolites profile after oral intake of blueberry juice, induced an increase of HO-1 and GCLM concentrations protecting from H_2_O_2_-induced oxidative stress [119].

Diabetes mellitus and metabolic syndrome are characterized by increased circulating levels of free fatty acids (FFAs) as a consequence of adipose tissue insulin resistance [129]. It is well documented the involvement of FFAs in the progression and enhancement of endothelial dysfunction [130]. Several recent studies found that Cy3-glc protects HUVECs against the deleterious actions of palmitic acid by inducing nuclear translocation of Nrf2 and a subsequent increase in intracellular GSH levels [131,132].

As previously stated, Nrf2 is also subject to regulation through Keap1-independent mechanisms, including phosphorylation and acetylation [133]. Mitogen-activated protein kinases (MAPK) and phosphatidylinositol-3-kinase/protein kinase B (PI3K/Akt) are two signaling pathways that lead to Nrf2 activation through phosphorylation. Protein kinase C (PKC) is also involved in the regulation of this transcriptional factor. Thus, Nrf2 integrates signals from different signaling pathways. There is evidence for the ability of ACN and their metabolites to modulate the aforementioned signaling pathways. For example, Chinese bayberry extract (rich source of Cy3-glc, Cy, cyanins, and Mv), protects pancreatic β cells against H_2_O_2_-induced cell injury [128]. Treatment of these cells with the extract induced activation of the MAPK and PI3K/Akt signaling pathways, as well as nuclear translocation of Nrf2, followed by HO-1 upregulation. Similar results were reported by another study which showed that human serum ACN and/or their metabolites protected HUVECs against the deleterious effects of mild hyperoxia (O_2_ 32%) [134]. Exposure to the human serum containing ACN and/or metabolites activated ERK1/2 kinases of the MAPK signaling pathway with subsequent activation of the Nrf2, leading to HO-1 and NQO1 upregulation. It was found that ferulic acid induces the antioxidant response in HUVECs through activation of of PI3K/Akt and ERK1/2 signaling pathways, followed by subsequent nuclear translocation of Nrf2 [114].

Oxidized LDL particles (oxLDL) exert a cytotoxic effect on the vascular endothelium through ROS production, impairment of NOS activity, and enhancement of pro-inflammatory genes expression [135]. Thus, oxLDL are involved in the progression of atherosclerosis. It was found that pretreatment of porcine aortic endothelial cells with De-3-glc offered protection against oxLDL-induced oxidative stress by reducing the levels of NADPH oxidase subunits Nox2, Nox4, and p22phox [136], while a more recent study proposed a different mechanism to explain the protective effect of De-3-glc on HUVECs exposed to oxLDL [137]. It was found that De-3-glc promoted AMP-activated protein kinase (AMPK) phosphorylation leading to sirtuin 1 (SIRT1) activation and subsequent stimulation of autophagy.

An important hallmark of the endothelial dysfunction is the decrease in NO^●^ synthesis and bioavailability with subsequent loss of the vascular tone [110]. It was shown that Cy3-glc activates the Src-ERK1/2 signaling pathway in bovine artery endothelial cells (BAECs) with subsequent activation of the specificity protein 1 (Sp1) transcription factor [138]. As a result, there was an enhancement of the endothelial NOS (eNOS) gene transcription leading to eNOS proteins synthesis and increased NO^●^ bioavailability. At the same time, activation of the Src-ERK1/2 pathway was found to be responsible for eNOS phosphorylation on Ser1179, but dephosphorylation at Ser116 of the same protein [139]. Phosphorylation on Ser1179 was followed by enhanced interaction with soluble guanylyl cylase and increased cyclic GMP production. It is important to mention that for the first study the authors used a physiologically relevant concentration of Cy3-glc (0.1 μmol/L). Taken together, these data clearly indicated a beneficial effect of Cy3-glc on the eNOS level and activity, with a positive impact on the vascular endothelium. A similar mechanism of action was described for De [140], which activates the ERK1/2 signaling pathway leading to increased eNOS expression which significantly reduced BAECs apoptosis elicited by actinomycin D and 7*β*-hydroxycholesterol. Mv3-glc (25 μM) was found to have a dual effect on NO^●^ production in BAECs [141]. Thus, Mv3-glc up-regulated eNOS mRNA leading to increased production of NO^●^, and down-regulated iNOS mRNA in cells stimulated with peroxynitrite anion.

Both Cy and De were found to decrease endothelin-1 (ET-1) synthesis in HUVECs, De being more active when compared to Cy [142]. These ACNDs induced a significant dose-dependent decrease on both protein and mRNA levels of ET-1. On the other hand, both compounds lead to an increase of eNOS protein level. Once again, De was more active than Cy. Thus, it appears that these ACNDs can decrease the concentration of the factor responsible for vasoconstriction with a concomitant increase in the concentration of the factor responsible for the opposite action. Pretreatment of HUVECs with Cy3-glc reversed the deleterious effects of palmitic acid by restoring the eNOS expression and NO^●^ synthesis [132]. A recent study found that ACNs from blackcurrant extract acted through direct interaction with estrogen receptors, leading to upregulation of the eNOS protein and NO^●^ synthesis [143].

Exposure of endothelial cells to different pro-inflammatory agents, like tumor necrosis factor-α (TNF-α), peroxynitrite anion, and oxLDL among others, is followed by nuclear factor kB (NF-kB) transcription factor activation through a canonical or a noncanonical (alternative) pathway [144]. In its inactive form, this transcription factor is kept in the cytoplasm as a complex with inhibitor of kB (IkB) protein. Stimulation of the canonical pathway leads to the activation of the IkB kinase (IKK) complex which triggers the phosphorylation of IkB leading to its ubiquitination and proteasomal degradation. Activated NF-kB dimers translocate to the nucleus inducing the transcription of genes coding for adhesion molecules (E-selectin, P-selectin, VCAM-1, ICAM-1), chemokines, growth factors, and inducible enzymes (iNOS, COX2), inducing an inflammatory response and promoting leukocytes adhesion.

Different studies indicated that the use of ACNs is associated with suppression of the oxLDL, cytokines (TNF-α) or cluster of differentiation 40 ligand (CD40L) activation of the NF-kB transcription factor leading to a correction of the pro-inflammatory phenotype of the endothelial cells [115,118,145]. Thus, it was found that De decreases oxLDL-induced expression of P-selectin and ICAM-1 with subsequent limitation of the adhesion of monocytes to the endothelial cells [145]. These results were obtained by using three different concentrations of De which are not physiologically relevant (50, 100, and 200 μM). ACN metabolites are also responsible for some of the anti-inflammatory effects [115]. Thus, it was found that Cy3-glc metabolites and not the parental compound were able to significantly reduce IL-6 and VCAM-1 in oxLDL-stimulated HUVECs. Protocatechuic acid-4-sulfate and ferulic acid were most active in reducing the levels of IL-6 and VCAM-1, respectively, at physiologically relevant concentrations (0.1, 1, and 10 μM) of ACN metabolites. Further evidence was provided for the fact that physiologically relevant concentrations (0.1–2 μM) of ACN metabolites lead to an inhibitions of monocytes adhesion to HUVECs [116]. On the other hand, the authors were not able to detect any significant effect on the expression of genes coding for E-selectin, ICAM-1, and VCAM-1, which suggest the involvement of unknown mechanisms. The correction of the pro-inflammatory phenotype of the endothelial cells is, at least in part, the result of the NF-kB activity attenuation [145].

A more recent study using two different mixtures of ACNs and ACN metabolites provided further insight into the mechanism of action of these compounds on the endothelium [102]. Thus, it was found that ACNs and their metabolites are able not only to inhibit the adhesion of monocytes to activated endothelial cells, but also to limit the chemokine-induced transendothelial migration of these cells which is an early step in the pathogenesis of the atherosclerotic plaque formation. Moreover, this study indicated that the two mixtures correct the pro-inflammatory phenotype of the endothelial cells through modulation of genes coding for proteins involved in intercellular adhesion (cadherin-5, CDH-5), migration of monocytes toward endothelium (C-X-C motif chemokine ligand 12, CXCL12), and adhesion and transendothelial passage (ICAM-1, integrin alpha-5).

Recently, modulation of microRNA expression emerged as a new mechanism of action for ACN [101]. Physiologically relevant concentrations of ACNs and ferulic acid modulate the expression of some miRNA species in the livers of ApoE-/- mice kept for two weeks on diets supplemented with different natural compounds. A recent study indicated that ACNs and their corresponding metabolites were able to modulate miRNA expression in TNF-α-stimulated HUVECs [102].

## 5. Evidence for Anthocyanins’ Effects in the Prevention and Treatment of Stroke

### 5.1. Epidemiological Studies

In the scientific community, there is a debate going on about the relationship between ACNs on CVD and stroke. A diet rich in fruits and vegetables is often associated with a reduced incidence of ischemic stroke, due to the high content of flavonoids, especially ACNs [146]. In a meta-study [147] on nineteen prospective cohorts it was demonstrated that the dietary anthocyanin level was in an inverse relationship with the risk of cardiovascular diseases and coronary heart disease, which is in accord with the other findings [148]. Also, the same study showed that there is no clear relationship between the anthocyanin dietary content and the risk of stroke, myocardial infarction or total cardiovascular diseases, albeit the dietary flavonoid was associated with reduced risk of stroke [8] and total cardiovascular diseases [149]. These contradictions could be the result of the fact that the ACN intake is likely to be more comprehensive than the intake of berries alone and of the difficulty of determination of the anthocyanin intake from the diet, their metabolic transformation, or even and their retention after cooking [150]. A compressive stratification by types of stroke, in terms of different mechanisms, revealed that there is no relationship between the dietary intake of ACNs and cerebral infarction, ischemic or hemorrhagic strokes [147]. These conclusions are in accord with other meta-studies that showed that an abundance diet in berries is not necessarily associated with the lower risk of stroke [151]. Another good explanation for the different results is the fact that ACN are not uniformly distributed in berries [152].

Even though there are studies that associate a high anthocyanin diet with a decrease risk of cardiovascular disease [153,154], the literature available till now which are related to stroke mention that there is no clear evidence for a protective effect of ACN uptake from different exogenous sources [155]. In a prospective cohort study (7091 total deaths, 2316 CVD deaths, 1329 CHD deaths, and 469 deaths due to stroke) on postmenopausal women, the relationship between a rich flavonoid diet and cardiovascular disease and stroke was evaluated [154], showing no clear correlation between reduced mortality associated with stroke and cardiovascular disease and a rich flavonoid diet, including ACNs.

### 5.2. In Vitro Studies

The main endogen excitatory neurotransmitter present in the mammalian central nervous system is glutamate. The toxicity of glutamate is an induced pathological process, in which cells are damaged and killed by excessive stimulation by glutamate and other similar compounds. This exo-toxicity leads to oxidative and nitrosative stress and contributes to the pathology of traumatic brain injury, stroke, neurodegenerative disorders, and normal brain ageing. Rat brain cultures were exposed to glutamate and while the blueberry extract was highly protective against glutamate toxicity, lingonberry fruits were not protective. At the same time, the extracts from leaves of both plants exerted a neuroprotective effect in the brain cell culture (glial cells composed mainly of astrocytes obtained from the brains of 1−3-day-old rat pups). This study suggests that high blueberries diets possibly slow brain ageing or inhibit the development of neurodegenerative disorders and stroke [156].

The neuroprotective effects of a black soybean seed coat extract (BSSCE) comparatively with purified Cy3-glc were investigated on a pure primary culture of cortical neurons from Sprague Dawley rats in which in vitro ischemia was simulated by exposing cells to oxygen-glucose deprivation and glutamate-induced cell death. By monitoring the glutamate-induced neurotoxicity, neuronal injury and intracellular ROS (reactive oxygen species) content, it was noted that BBSCE could prevent membrane damage in a dose-dependent manner, at the same time increasing the viability of primary neurons that were exposed to oxygen-glucose deprivation. Surprisingly, black soybean ACNs were not able to protect against glutamate-induced neuronal cell death, but rather inhibited the excessive generation of ROS, thus preserving the mitochondrial membrane potential in primary neurons exposed to oxygen-glucose deprivation. These results suggested that the neuroprotective activity of soybean extracts and of Cy3-glc is associated with oxidative stress inhibition and mitochondrial membrane potential, but not with glutamate-induced neuronal cell death [157].

The term hypoxia refers to oxygen levels below normal (arterial oxygen pressure is 11.0–14.4 kPa, arterial oxygen saturation is 95–98%) [158], and the adaptative response to hypoxia is mediated by the activation of the hypoxia-inducible factor-1 (HIF-1) which regulates the expression of different genes coding for proteins that help astrocytes to cope with the low level of oxygen. Moreover, several other signaling pathways are activated by hypoxia, including NF-kB, p53, cAMP, CREB and c-jun [159].

Astrocytes are the most abundant cell type in the central nervous system and their activity is affected by hypoxia despite the fact that they have a greater capacity to cope with the stress when compared to neurons [160]. A recent study provided an insight into the regulation of gene expression in primary human cortical astrocytes cultured under both hypoxia and hypothermia [161]. Using human gene expression microarrays researchers identified the up- and downregulated genes. Some of these signaling pathways lead to an increased production of proinflammatory cytokines IL-6, TNFα, IL-1α and β, interferon γ, among others, which can be detrimental to ischemic recovery [162]. On the other hand, activated astrocytes release glutathione and SOD which could play an important role in neuronal survival following hypoxia.

Astrocytes are a type of glial cells which helps in maintaining optimal environment for neuronal functions in CNS. They have a modulatory role on neuropathological events as they are a reservoir for antioxidants and release essential neurotrophic factors. Failure of astrocytes functions or altering their viability lead to neuronal degeneration and disruption, associated with inflammation and infarction volume after stroke, in mouse models. Some recent studies revealed that ACNs can regulate the expression of the genes involved in atherosclerosis and induce apoptosis or autophagy [163]. These findings suggested a potential role of anthocyanin in the regulation of signal pathways involved in inflammation and apoptosis upon exposure to oxidative stress.

Black soybean extracts were analysed regarding the survival of U87 glioma cells under oxygen-glucose deprivation, which mimics the ischemic condition in vivo. The cells were exposed to oxidative stress conditions in the presence or absence of anthocyanin extract (up to 100 μg/mL) and an increase of cell viability in the presence of ACNs was observed, in a dose-dependent manner. The increase in viability was associated with decreased ROS levels. Also, in the cells that underwent pre-treatment with ACNs from black soybeans cells, the induction of autophagy was recorded under hypoxic condition, by activation of the autophagosome marker LC3 and conferring protection on U87 cells, albeit the mechanism could be not directly associated with ROS levels [164].

Mulberries (*Morus alba* L.) extracts have high anthocyanin content, with Cy3-glc (chrysanthemin) the most abundant. The neuroprotective effect of the mulberry extract was evaluated on mouse cerebral PC12 cells, which were exposed at oxidative stress induced by H_2_O_2_. The mulberry fruit extract inhibited the cerebral ischemic damage caused by oxygen glucose deprivation in PC12 cells. The neuroprotective effect of the mulberries extract and its most important component, Cy3-glc on PC12 cells was dose-dependent in terms of viability and myeloperoxidase immunohistochemistry [165].

Blueberry extract was analysed on the lipopolysaccharide LPS-activated murine BV2 microglia cell line because this model system mimics the microglial responses. The treatment with blueberry extracts lead to a decrease in activation of pro-inflammatory mediators in BV2 cells as result of: inhibition of nitric oxide (NO) production, reduction of the levels of cytokines in complete culture medium (CCM), the decrease of protein expression of iNOS and COX-2 proteins levels and decrease in the intracellular ROS production. Blueberry extract was found to inhibit the neuroinflammation, by inhibiting the production of NO, IL-1β and TNF-α in microglia cells [166].

The ability of Cy3-glc and its in vivo metabolites—Cy and protocatechuic acid—to exert a neuroprotective effect was evaluated on the human neuronal cell line (SH-SY5Y) exposed to the oxidative stress induced by H_2_O_2_. In the same study, the protective effect of this compound against ROS formation and apoptotic events in mitochondria were also monitored, because the intracellular accumulation of ROS and consequently oxidative stress lead to neuronal apoptosis. The antioxidant pre-treatment of SH-SY5Y cells revealed specific inhibitory action against ROS production at different cellular levels: Cy3-glc at the membrane level, protocatechuic acid at cytosol level and Cy at both cellular levels, higher than both Cy3-glc and protocatechuic acid. Cy and protocatechuic acid, but not Cy3-glc, can inhibit H_2_O_2_-induced apoptotic events in mitochondria probably due to different bioavailability [167].

In spite of the interesting results obtained so far, caution is needed when drawing any conclusion regarding the neuroprotective action of ACNs, as the same authors [167] mentioned that in vivo Cy3-glc and its metabolites at low concentrations (<25 µM) exhibit no clear neuroprotective activity. The apoptotic effect of protocatechuic acid was also reported on a study using protocatechuic acid isolated from *Alpinia oxyphylla,* whose neuroprotective effect was assessed on PC12 cells and aged rats [168].

### 5.3. In Vivo Studies

#### 5.3.1. Clinical Studies on Animals

● Studies related to stroke prevention

Experimental animal models that are associated with risk factors for stroke mainly target hypertension [169], atherosclerosis [170], hypercholesterolemia [171], obesity and aging [172]. A well-known experimental model for stroke studies is represented by spontaneously hypertensive stroke-prone rat (SHRSP), a model which is suitable for large artery stroke [173], and there are several studies in which the effects of ACN-containing diets on SHRSP compared with normotensive rats were assessed. Such studies suggested that a diet supplemented with blueberry can reduce systolic blood pressure in SHRSP rats compared with the normotensive rats [174], and this effect could be a consequence of angiotensin-converting enzyme (ACE) inhibition [169]. On the other hand, it was found that supplementation of the mice or rats’ diet with blueberries extracts led to the increase of short-term memory and the motricity [175]. Also, this type of supplements administrated to 19-month-old rats for 8 weeks lead to the reversing the course of neuronal and behavioral aging [176].

● The effects of ACNs on induced stroke

In humans, ischemic stroke has an incidence around 80% of the total number of cases [177]. For this reason, most studies that investigated the effects of ACNs or anthocyanin-rich plant extracts on animals undergoing strokes were accomplished using experimental models that induced this type of stroke. Several methods are described for inducing ischemic vascular accidents in in vivo studies conducted on different types of laboratory animals. Cerebral ischemia patterns in animals were classified as global and focal models, which reduce cerebral blood flow in the entire brain or in a certain brain areas [178]. It is thought that focal patterns are more relevant to stroke accidents in humans and they are often performed on the middle cerebral artery (MCA) and may be transient or permanent [179]. Both models of ischemia induction (global and focal) were applied in studies on the effects of ACN administration. Some studies reported their beneficial effect when administered before stroke induction [82,180,181,182,183,184], others after the stroke induction [165,185,186], while a third set of studies monitored the effects of ACN administration before and after stroke induction [81,187,188,189].

● Administration of ACNs after stroke induction

In a recent study, a transient ischemic stroke was induced for 2 h by ligating common carotid artery (CCS), external carotid artery (ECA), and internal carotid artery (ICA) on proximal MCA. After releasing arteries and reperfusion, a Balinese cultivates purple potato extract, which contained 209.8 mg anthocyanin/100 g, was administrated in Wistar rats. In the treatment group a higher level of BDNF (brain-derived neurotrophic factor) was found, while apoptosis induction factor (AIF) was significantly reduced. Moreover, the animals from this group had a better neurological score than those from the control group [185]. In another study with Cy3-glc and mulberry extract, a significant decrease of 18% and 26%, respectively, was obtained [165]. A reduced number of myeloperoxidase positive cells was also reported. In this case the cerebral ischemia was accomplished by the MCA transient occlusion (90 min) using intraluminal filament technic in mice. A similar method was applied using Provinols, with a much more complex composition (61 g/kg total ACN, 19 g/kg free ACN, and important amounts of proanthocyanidins, polymeric tannins, catechin, hydroxycinnamic acids, and flavonols, in saline solution), in male Wistar rats [186]. It was found that, after a single bolus (0.1 mg/kg, i.v.), the lactate levels were massively reduced in the group that received Provinols (during reperfusion), while the glucose and lactate concentration had been previously raised (during occlusion). This result could not be attributed exclusively to the presence of ACN, and it was evident that Provinol improved the use of lactate as a substrate for the survival of neurons; other parameters also suggest its positive effects on stroke (reducing taurine, aspartate and amino acids glutamate).

● Administration of ACNs before stroke induction

In experimental models the brain infarct volume and the apoptotic neurons provoked by induced stroke followed by reperfusion could be reduced by previous administration of ACNs [183]. Studies regarding the effect of pre-administration of ACNs on stroke used purified ACNs [82,181,182] or even encapsulated ACNs available on the market [183] (Table 4) with the aim to elucidate the molecular mechanisms by which ACNs act (see Section 4). Two modalities of stroke induction were applied, most often used is MCA occlusion model of focal ischemia [82,180,181,182,183] and only a single study used bilateral common carotid artery occlusion (BCCAO), which is a model of global ischemia [184], while various ways to administer ACNs are applied (Table 4). In most experimental models, laboratory animals were tracked and subsequently sacrificed 24 h after reperfusion [82,182,183] and only in one study after 72 h [184]. A comparison between 24 h and 72 h after reperfusion was only revealed by Dewi [180]. Rats were the most used laboratory animals in these studies [82,181,183,184] and less often mice [182]. On the other hand, there is a wide variety in the parameters monitored in these latter studies (neurological, biochemical, histopathological, etc.). The results obtained so far show that pre-administration of ACNs in induced stroke reduced volume infarction [82,182,183] by triphenyltetrazolium chloride (TTC) or cresyl violet (CV) staining methods.

It was determined that ACNs also inhibit apoptosis rate, measured by TUNEL assay (TdT-medicated dUTP-Nick End Labeling assay) [181,183] and two studies reported that ACNs could have anti-apoptotic activities, as shown by the increase of Bcl-2 (B-cell lymphoma) expression protein, which is known for the involvement in apoptosis regulation [82,181]. A reduction of apoptosis has been recently reported in cerebellum [180] by an immunohistochemistry approach, using an in situ apoptotic detection method.

Other positive effects of anthocyanin pre-treatment were also mentioned in induced stroke, such as improvement of spontaneous activity and memory [183,184] and reduction of molecule levels involved in inflammatory answer (tumor necrosis factor-a (TNF-a), interleukin-1b (IL-1b), and interleukin-6 related with) [82]. The reduction of myeloperoxidase (MPO) activity in brain was also found [184]. In another study, the macroscopic and microscopic observation of the brain tissue treated with purified extract of ACNs from bayberry (*Myrica rubra*, specially Boqi variety) which predominantly contains Cy3-glc (up to 95%) showed beneficial protective effect against cerebral ischemia-reperfusion; this protective effect could be associated with the Toll-like receptor 4 (TLR4)/nuclear factor-κB (NF-κB) TLR4/NF-κB, NOD-like receptor pyrin domain-containing 3 protein (NLRP3) and Nrf2/antioxidant responsive element pathways. It was demonstrated that malonyl dialdehyde (MDA) amounts were lower and superoxide dismutase (SOD) activity was improved in treated mice with ACNs in comparison with control group [182].

A remarkable study was carried out by administering for 2, 4 and 6 months an extract of *Vaccinum myrtillus*, (Table 4) in golden hamsters with BCCAO (for 30 min) followed by reperfusion (60 min). The ROS, damage area, microvascular leakage, leukocyte adhesion and capillary perfusion were reduced in fed-hamsters with extract by comparison to controls [191].

● Administration of ACNs before and after stroke induction

The administration of ACNs before and after induction of ischemic stroke was done using the same experimental models, on different animal groups, in mice [188] or in rats [187]. In the two studies Cy3-glc was administrated by oral gavage [188] or i.p. [187], while the ischemic strokes were induced by permanent middle cerebral artery occlusion [188] (pMCAO) or by BCCAO [187], respectively. Pretreated and post-treated rats with 2 mg Cy3-glc/kg bw showed reduced infarction volume [188]. These have revealed that both types of ACN administration could be useful for prevention as well as for treatment of ischemic brain damage. On the other hand, the administration of Cy3-glc resulted in an improvement of heme- oxygenase and γ-glutamylcysteine synthase expression in brain tissue of mice treated compared to the control group. Significant decrease in the level of lipid hydroperoxides was also reported, while the non-proteic thiol increased in the brain homogenate [187].

Two studies investigated the effects of ACND pre-treatment and post-treatment on stroke induced in the same rats [81,189], indicating that neuronal damages were attenuated in the rat brains with induced stroke (by 30 min BCCAO) and that received Mv and Cy, respectively. It was also found that Cy and Mv acted against vessel wall damage in the rat brains by diminishing leukocyte adhesion and microvascular leakage as well as they reduce ROS production (by using 2′,7′-dichlorofluorescein-diacetate assay) [81,189]. An unexpected result was obtained for Cy-treated group when a greater reduction in infarct size was recorded at a lower dose [189].

#### 5.3.2. Clinical Studies in Humans

It was proved that blueberries as well as cranberries increase flow-mediated dilation (a common technique used for assessing endothelial function) in healthy adults, after intake of a high quantity of polyphenols [192,193]. A significant increase in flow-mediated dilation was found following consumption of a freeze-dried grape preparation containing ACNs for 30 days by men with metabolic syndrome [194] but no effect of grape wine (800 mg total polyphenols) on flow-mediated dilation was observed over a three-week of administration to healthy males [195]. These contradictory results suggest the ability of ACN to produce effects only when they are present in the blood flow, so for an optimum effect, the anthocyanin administration would be distributed evenly during the day [196].

The feeding on blueberries for 12 weeks improved cerebral blood flow on parietal and occipital lobe, in healthy older adults [197], and at the same time, it was proved that higher uptake of ACNs led to a 12% decrease of hypertension, one of the risk factors for ischemic stroke [198]. This finding was also confirmed by a study which enrolled 24 adults (12 elder and 12 younger) in an acute cross-over study [199]. Thus, it was found that ingestion of 300 mL plum juice with high content of ACNs significantly reduced blood pressure in both groups, and more visibly in the older patients. An important reduction in blood pressure in hypertensive patients was also observed [200] when the subjects received treatment for 4 weeks with an extract obtained from *Hibiscus sabdariffa*, containing 250 mg total ACNs.

Purple potatoes seem also to have effective hypotensive action in hypertensive patients [201]. As a consequence, it is expected that ACN consumption would lead to a lower stroke risk. However, the administration of high amounts of ACNs for long periods of time for stroke prevention does not seem justified in the light of recent results of the epidemiological studies (see Section 3.1.).

## 6. Conclusions

There are no clear data on the possible synergic effects of ACNs with their metabolites or with other bioactive compounds; nevertheless it is now accepted that ACNs sometimes act through their metabolites [36]. Moreover, there are studies that testify to the neuroprotective effects of or against ischemia-induced oxidative damage of ACN metabolites such as phenolic acids: vanillic acid [202], ferulic acid [203] and syringic acid [204].

In addition, there are studies that refer to the administration of plant extracts with ACNs that are not completely characterized. The effect of the food matrix as a whole and the presence of certain nutrients could also contribute—both positively and negatively—to certain effects associated with the bioavailability of ACNs. On the other hand, despite the modern techniques used to quantify metabolites and anthocyanin degradation products in various biological samples, the commercial unavailability of some standard compounds is still an impediment to the fully understanding of metabolic processes in which ACNs are involved [60].

Taken together, the studies mentioned above provide evidence for the beneficial effect of ACNs and their metabolites for the vascular endothelium biology. Through direct and indirect mechanisms, these compounds have the potential to limit and counteract the action of some factors that have deleterious effect on endothelial cells. These mechanisms could be exploited for the use of these compounds in the prevention of cardiovascular disease, including stroke.

## Figures and Tables

**Table 1:** The main anthocyanidins (ACND) and anthocyanins (ACN) from the human diet and the richest sources.

ACND/ACN (Abbreviation)	R1	R2	R3	R4	Latin Name of Plant Sources	Main Sources	Mean Content mg/100 g	Reference
Pelargonidin (Pg)	-H	-H	-OH	-OH	*Fragaria* sp. *Phaseolus vulgaris* *Camellia sinensis*	Strawberry Common bean (black) Tea (Kenyan purple leaf)	4.31 0.95 0.841	[14] [14] [15]
Cyanidin (Cy)	-OH	-H	-OH	-OH	*Camellia sinensis* *Phaseolus vulgaris* *Rubus idaeus* *Fragaria* sp.	Tea (Kenyan purple leaf) Common bean (black) Red raspberry Strawberry	1.751 1.63 0.53 0.50	[15] [14] [14] [14]
Delphinidin (De)	-OH	-OH	-OH	-OH	*Camellia sinensis*	Tea (Kenyan purple leaf)	0.121	[15]
Peonidin (Pn)	-OCH3	-H	-OH	-OH	*Phaseolus vulgaris*	Common bean (black)	1.36	[14]
Petunidin (Pt)	-OCH3	-OH	-OH	-OH				
Malvidin (Mv)	-OCH3	-OCH3	-OH	-OH	*Camellia sinensis*	Tea (Kenyan purple leaf)	0.301	[15]
Pelargonidin-3-glucoside (Pg3-glc)	-H	-H	glc	-OH	*Fragaria* sp. *Phaseolus vulgaris* *Morus nigra*	Strawberry Common bean (black) Mulberry	47.14 12.60 27.8 ± 2.1	[14] [14] [16]
Pelargonidin-3-rutinoside (Pg3-rut)	-H	-H	rut	-OH	*Ribes nigrum* *Morus nigra*	Blackcurrant Mulberry	2.48 1.4 ± 0.2	[14] [16]
Pelargonidin-3-(6′′-succinyl-glucoside) (Pg3-(6′′-succ-glc)	-H	-H	6′′-succ-glc	-OH	*Fragaria* sp.	Strawberry	10.44	[14]
Cyanidin-3-glucoside (Cy3-glc)	-OH	-H	glc	-OH	*Aronia melanocarpa* *Sambucus nigra* *Rubus fruticosus* ^2^ *Ribes nigrum* *Prunus domestica* *Rubus idaeus* *Ribes rubrum* *Prunus avium* *Phaseolus vulgaris* *Oryza sativa* ^3^ *Morus nigra*	Black chokeberry Black elderberry Blackberry Blackcurrant Plum Red raspberry Redcurrant Sweet cherry Common bean (black) Black rice (some varieties) ^3^ Mulberry	19.64 797.13 138.72 25.07 8.63 14.89 3.37 18.73 3.99 0–470 704.1 ± 20.4	[14] [14] [14] [14] [14] [14] [14] [14] [14] [17] [16]
Cyanidin-3-galactoside (Cy3-gal)	-OH	-H	gal	-OH	*Aronia melanocarpa* *Vaccinium vitis-idaea* *Malus domestica*	Black chokeberry Lingonberry Apple	557.67 48.69 0.81	[14] [14] [14]
Cyanidin-3-rutinoside (Cy3-rut)	-OH	-H	rut	-OH	*Rubus fruticosus* ^2^ *Ribes nigrum* *Prunus domestica* *Rubus idaeus* *Ribes rubrum* *Prunus cerasus* *Prunus avium* *Morus nigra*	Blackberry Blackcurrant Plum Red raspberry Redcurrant Sour cherry Sweet cherry Mulberry	8.86 160.78 33.85 5.20 2.10 6.98 143.27 572.1 ± 22.5	[14] [14] [14] [14] [14] [14] [14] [16]
Cyanidin-3-sambubioside (Cy3-samb)	-OH	-H	samb	-OH	*Sambucus nigra* *Ribes rubrum*	Black elderberry Redcurrant	462.96 9.47	[14] [14]
Cyanidin-3-sophoroside (Cy3-soph)	-OH	-H	soph	-OH	*Rubus idaeus* *Ribes rubrum*	Red raspberry Redcurrant	37.61 2.62	[14] [14]
Cyanidin-3-arabinoside (Cy3-ara)	-OH	-H	ara	-OH	*Aronia melanocarpa* *Sambucus nigra* *Vaccinium vitis-idaea*	Black chokeberry Black elderberry Lingonberry	252.76 252.76 5.85	[14] [14] [14]
Cyanidin-3-xyloside (Cy3-xyl)	-OH	-H	xyl	-OH	*Aronia melanocarpa* *Rubus fruticosus* ^2^	Black chokeberry Blackberry	45.90 9.74	[14] [14]
Cyanidin-3- glucosyl-rutinoside (Cy3-glc-rut)	-OH	-H	glc-rut	-OH	*Rubus idaeus* *Ribes rubrum* *Prunus cerasus*	Red raspberry Redcurrant Sour cherry	7.06 4.23 43.63	[14] [14] [14]
Cyanidin-3- xylosyl-rutinoside (Cy3,5-xyl-rut)	-OH	-H	xyl-rut	-OH	*Ribes rubrum*	Redcurrant	11.22	[14]
Cyanidin-3,5-diglucoside (Cy3,5-diglc)	-OH	-H	glc	glc	*Sambucus nigra*	Black elderberry	17.46	[14]
Delphinidin-3-glucoside (De3-glc)	-OH	-OH	glc	-OH	*Ribes nigrum* *Vitis vinifera* *Vaccinium augustifolium* *Phaseolus vulgaris*	Blackcurrant Grape (black) Lowbush blueberry Common bean (black)	86.68 2.63 15.17 14.50	[14] [14] [14] [14]
Delphinidin-3-galactoside (De3-gal)	-OH	-OH	gal	-OH	*Vaccinium augustifolium*	Lowbush blueberry	16.14	[14]
Delphinidin-3-rutinoside (De3-rut)	-OH	-OH	rut	-OH	*Ribes nigrum*	Blackcurrant	304.91	[14]
Delphinidin-3-glucosyl-glucoside (De3-glc-glc)	-OH	-OH	glc-glc	-OH	*Allium cepa* L. *var. cepa*	Onion (red)	6.50	[14]
Peonidin 3-glucoside (Pn3-glc)	-OCH3	-H	glc	-OH	*Vitis vinifera* *Vaccinium vitis-idaea*	Grape (black) Lingonberry	5.80 4.25	[14] [14]
Peonidin 3-rutinoside (Pn3-glc)	-OCH_3_	-H	rut	-OH	*Prunus domestica* *Prunus cerasus* *Prunus avium*	Plum Sour cherry Sweet cherry	4.85 2.70 7.42	[14] [14] [14]
Petunidin-3-glucoside (Pt3-glc)	-OCH_3_	-OH	glc	-OH	*Vaccinium augustifolium* *Oryza sativa* ^3^	Lowbush blueberry Black rice (some varieties) ^3^	11.20 0–40	[14] [17]
Malvidin 3-glucoside (Mv3-glc)	-OCH_3_	-OCH_3_	glc	-OH	*Vitis vinifera* *Vaccinium augustifolium* *Vitis vinifera*	Grape (black) Lowbush blueberry Red wine from grape	39.23 26.06 9.97 ^(1)^	[14] [14] [14]
Malvidin 3-galactoside (Mv3-gal)	-OCH_3_	-OCH_3_	gal	-OH	*Vaccinium augustifolium*	Lowbush blueberry	21.43	[14]
Malvidin 3-*O*-(6′′-acetyl-glucoside) (Mv3-(6′′-Ac-glc)	-OCH_3_	-OCH_3_	6′′-Ac-glc	-OH	*Vitis vinifera* *Vaccinium augustifolium* *Vitis vinifera*	Grape (black) Lowbush blueberry Red wine from grape	9.66 14.74 3.52 ^(1)^	[14] [14] [14]

ACND: Anthocyanidins; ACN:anthocyanins; glc: glucosyl/glucoside; gal: galactosyl/galactoside; ara: arabinosyl/arabinoside; rut: rutinosyl/rutinoside; samb: sambubiosyl/sambubioside; soph: sophorosyl/sophoroside. ^(1)^ amount in mg/mL; ^2^ *Rubus allegheniensis*, native to eastern North America, *Rubus fruticosus*, native to Europe; ^3^ *Oryza sativa* cv. Heugjinjubyeo.

**Table 2:** The evaluation of interaction between the anthocyanins (ACN), and some of their metabolites and the blood–brain barrier (BBB): in vitro studies.

ACN or Metabolite	Final Concentration of Compound before Incubation (µM)	Transport Efficiency/Uptake/Recovery	Method Applied	References
Cy3-glc	100	11.4 ± 2.6, 1 h; 13.72 ± 2.66, 3 h; 21.07 ± 5.2, 18 h ^1^	HPLC-DAD	[71]
Cy3-glc in presence of 0.1% ethanol	100	5.99 ± 1.18, 1 h; 13.1 ± 0.68, 3 h; 17.8 ± 0.31, 18 h ^1^	HPLC-DAD	
De3-glc	100	5.0 ± 0.7, 1 h; 8.8 ± 1.1, 3 h; 11.6 ± 0.6, 18 h ^2^	HPLC-DAD/MS	[69]
4′Me-De3-glc	100	5.5 ± 1.3, 1 h; 11.5 ± 1.0, 3 h; 17.6 ± 1.7, 18 h ^2^	HPLC-DAD/MS	
Cy3-glc	100	8.0 ± 1.1, 1 h; 12.6 ± 0.9, 3 h; 16.0 ± 0.6, 18 h ^2^	HPLC-DAD/MS	
4′/3′Me-Cy3-glc	100	9.2 ± 2.0, 1 h; 13.4 ± 1.2, 3 h; 19.0 ± 1.4, 18 h ^2^	HPLC-DAD/MS	
Mv3-glc	100	5.3 ± 0.1, 1 h; 13.3 ± 2.4, 3 h; 20.0 ± 3.3, 18 h ^2^	HPLC-DAD/MS	
Cy3-rut	30	n.d., 2 h; 5.1±6.5, 6 h; 25.6 ± 8.5 18 h ^3^ n.d., 2 h and 6 h; 18.7 ± 3.2, 18 h ^4^	LC/MS-MS	[73]
Pg3-glc	30	n.d., 2 h; 9.9 ± 3.2, 6 h; 18.1 ± 6.6, 18 h ^3^ n.d., 2 h and 6 h; 11.23 ± 4.3, 18 h ^4^	LC/MS-MS	
Cy3-rut	30	83.2 ± 3.1 ^5^	LC/MS-MS	
Pg3-glc	30	84.3 ± 6.6 ^5^	LC/MS-MS	

HPLC, High-Performance Liquid Chromatography; DAD, Diode-Array Detection; MS, Mass Spectrometry; LC, Liquid Chromatography; ^1^ Transport efficiency (%; mean ± SEM), calculated based on (ACN or metabolite concentrations on the basolateral side at a given time)/(ACN or metabolite concentrations on the apical side at the zero hour) × 100; data extracted from figure. ^2^ Transport efficiency (%; mean ± SEM), calculated based on (ACN or metabolite concentrations on the basolateral side at a given time)/(ACN or metabolite concentrations on the apical side at the zero hour)x100. ^3^ b.END5 uptake (ng ACN/mg protein; mean ± SD); n.d., not detectable. ^4^ RBE4 uptake (ngACN/mg protein; mean ± SD); n.d., not detectable. ^5^ Recovery (% of initial dose recovered in donor plus receiver solutions) of ACN in the model of the BBB formed by ECV304 cells (human cell line expressing an endothelial phenotype) co-cultured with rat glioma cells (C6).

**Table 3:** The in vivo studies reporting anthocyanins (ACN) in brain tissue (parent compounds or metabolites) [89].

Source Composition	Dose Administrated, Administration Route	ACN/Metabolites in Brain Tissue	Method Applied	Experimental Model	References
Powdered blueberry 2% (w/w) in diet supplemented ^1^	ad libitum in food, 8 weeks, p.o.	Mv3-glc: 279 fmol/g cortex tissue, 432 fmol/g midbrain and diencephalon tissue ^2^	LC-MS/MS	15 healthy neutered 32–41-day-oldmale pigs (Yorkshire Landrace)	[46]
Powdered whole blueberry fruit (with 7.97 mg Cy3-glc equivalents/g) ^3^	ad libitum in food, 4 weeks, 0, 10, 20, 40 g/kg diet, 4 groups (around 1.48 mmol ANCs of dose/kg bw/day)	0.81 pmoli Cy3-glc equivalents/g FW (cortex) 0.36 pmoli Cy3-glc\equivalents/g FW (cerebellum)	LC-MS/MS	20 healthy neutered 32–41-day-oldmale pigs (Yorkshire Landrace)	[64]
Bilberry extract contained, (as mg aglycone/g extract): 69.69% De3-glc and others ^4^	82.5 mg/kg bw/day, 3 weeks, p.o.	Mv3-glc: 4.43 pmol/g ^5^	LC/MS-MS	40 commercial, newly weaned piglets	[87]
Blackberry extract with 47.9% ACN, Cy3-glc (91.2%)	14.8 mmol ACN/kg diet (with 13.5 mmol of Cy3-glc/kg diet/day) ad libitum, 15 days, p.o.	Cy3-glc: 0.21 ± 0.05 nmol/g tissue (84.0%)	HPLC-ESI-MS-MS and HPLC-DAD	12 male Wistar rats, (Iffa-Credo, L’Arbresle, France)	[90]
Tart cherry ACN 10% ^6^	20 g of diet powder/animal/day; approximately 10% below ad libitum intake, 3 weeks, p.o.	Cy3-glc-rut: 654.86 ± 3 2.18 fmoli/g tissue; Pn3-rut: 33.30 ± 2.95 fmoli/g tissue	LC/MS-MS	18 male Wistar rats, 6 weeks old	[91]
Kenyan purple tea contained: Cy, the most abundant (1755.60 μg/m), Pn, Pg, De, Mv	200 mg/kg bw/day, 14 days, p.o.	Identified: De, Cy, Pg, Pn, Mv	HPLC	15 healthy Swiss white mice, 8-week-old female adult	[15]
Pelargonidin (99.82 %), dissolved in 50% aqueous ethanol	50 mg pelargonidin/kg bw (one dose), p.o.	0.16 nmol pelargonidin/g tissue (2 h after administration), only compound detected	HPLC and LC-MS	6 male Sprague-Dawley rats	[67]
Pure ACN mixture extracted from *Vitis vinifera* grapes (almost 50% Mv3-glc)	8 mg/kg bw for 10 min, intragastrically administrated	Mv3-glc: 122.0 ± 54.72 pmoli/g; Mv3-(6-*O*-*p*-coumaroyl) glc: 196.54 ± 71.92 pmoli/g	HPLC-DAD-MS	13 male Wistar rats (Harlan Teklad 2018)	[92]
Cy3-glc (in PBS)	668 nmol (one dose), i.v.	Cy3-glc (pmol/g): 7.48±0.79 (2 min); 2.18 ± 0.58 (15 min); Pn3-glc (pmol/g): 2.07 ± 1.18 (2 min), 0.40 ± 0.38 (15 min); Pt3-glc (pmol/g): 1.15 ± 0.62 (2 min), 2.45 ± 0.22 (15 min)	UPLC/MS-MS	22 male Wistar rats (*Rattus Norvegicus*, Harlan Italy S.r.l.), same age (15 weeks)	[93]
2.7 μmol of polyphenol microbial metabolites (inclusive of ACN metabolites), dissolved in 30 μL methanol into 0.3 mL PBS (one dose) ^7^	HBA, a metabolite of Pg B-ring: 347; VA, a metabolite of Pn B-ring: 174; PCA, a metabolite of Cy B-ring: 226; GA, a metabolite of De B-ring: 3125, i.v. ^8^	HBA: 126.63 (control); 206.97 (2 min); 407.92 (15 min); VA: 99.71 (control); n.d. (2 min); 384.71 (15 min); PCA: n.d. (2 min), n.d. (15 min); GA: 612.72 (2 min); 610.82 (15 min) ^9^	UPLC/MS-MS	20 male Wistar rats (*Rattus Norvegicus*, Harlan Italy S.r.l.), same age (12 weeks)	[94]

^1^ The main ACN: Mv3-glc, Mv3-gal, Mv3-ara, De3-glc, Cy3-glc, Pt3-gal, Pt3-glc, Pt3-ara. ^2^ There were identified 15 anthocyanins: after Mv3-glc, the next most prevalent in all brain tissue: Mv3-gal, Pn3-glc, and Pt3-glc. ^3^ The main ACN (as *μg* of ACN/g of bilberry powder): 2631 De3-glc, 2524 De3-gal, 1046 Mv3-ara. ACNs also present: Cy3-gal, Cy3-glc, Cy3-ara, Mv3-glc, Mv3-gal, Pn3-gal. ^4^ Other ACN are: De3-gal 26.09%, De3-ara 19.05, Pe3-glc 10.58%, Pe3- gal 0.79, Pe3-glc 4.71, Pe3-ara 0.97, Mv3-gal 2.58, Mv3- glc 8.04, Mv3- ara 2.52, Pt3-gal 3.53, Pe3- ara 2.81, Cy3-gal 2.62, Cy3-glc 5.43, Cy3-ara 3.18 (as mg aglycone/g extract). ^5^ Total amounts obtained from cerebellum, medial frontal cortex and brain stem for Mv3-glc (the highest value among ACN)—data extracted from figure; ^6^ Cy3-glc-rut 325.9 ± 57.2 42, Cy3-rut 5-glc 120.2 ± 20.5; Cy3-rut 274.2 ± 43.2; Pn3-rut 40.4 ± 7.7; Pn3-glc 7.4 ± 1.1; Cy3-glc 3.1 ± 0.7; Cy3-sophoroside 2.8 ± 0. 5; Pg1.2 ± 0.4. ^7^ The phenols concentrations were in the concentration range noted in humans after the ingestion a standard serving of berry fruits. ^8^ Concentration expressed in pmol/g bw; ^9^ Concentration expressed in pmol/g FW, fresh weight; n.d., not detected; bw, body weight; HBA, 4-hydroxybenzoic acid; VA, vanillic acid; PCA, protocatechuic acid; GA, gallic acid, PBS, phosphate buffer solution; (2 min) or (15 min), represent time after administration; pelargonidin (Pg); cyanidin (Cy); petunidin (Pt); delphinidin (De); peonidin (Pn); malvidin (Mv).

**Table 4:** Anthocyanins used in studies with experimental models of induced stroke.

Anthocyanin	Sources	Reference
Total ACN: 147.0 mg/mL (method not specified)	Purple sweet potato, Balinese cultivar *Ipomoea batatas* L. (aqueous extracts, 1:1, kg/L)	[185]
Not specified	Purple sweet potato, Balinese cultivar *Ipomoea batatas* L. (aqueous extracts, 1:1)	[181]
Pt3,5-diglc, Pn3-glc, Mv3-glc, De3-glc, De3-(6-*O*-coumaroyl) glc (identified) ^1^	Purple sweet potato extracts, from Bali, *Ipomoea batatas* L. (extract anthocyanin, commercially available)	[180]
Cy3-glc (extracted and purified anthocyanin), purity 98.3%	*Morus alba* berries (mulberries)	[165]
Cy3-glc: 0–21.28% ^2^ Total ACN: 0–22.07% ^3^	*Myrica rubra* berries (bayberry) eight cultivars (Boqi 1, Boqi 2, Tanmei, Shuijing, Dongqui, Dingdai, Wandao, Wild) from China (purified anthocyanin extracts)	[182]
(Pt3-(*p*-coumaroyl)-rut-5-glc), purity 98.3%	Fruits of *Lycium ruthenicu* (extracted and purified anthocyanin)	[82]
Not specified	*Vaccinium angustifolium* (fresh lowbush blueberries)	[13]
Total ACN: 3.1% ^3^	Petals of *Echium amoenum* (total anthocyanin extract dried by lyophilization)	[184]
Flavonoids, ACN and phenolic acid compounds with antioxidant activity (no other data)	*Dorema aucheri* leaves (extract hidroalcoholic in ethanol 70%)	[190]
Total ACN: 34.7%, expressed as Cy3-glc	Fruits of *Vaccinium myrtillus* (lyophilized extract)	[191]
Medox-75 mg ACN/capsule	Concentrate from wild Scandinavian bilberries (*Vaccinium* sp.) and black currants (*Ribes nigrum*), commercially available	[183]
Provinols™ composition (in g/kg of dry powder): total ACN: 61; total ACND: 19; proanthocyanidins: 480; catechin: 38, hydroxycinnamic acids: 18, flavonols: 14, polymeric tannins: 3704	Red wine with polyphenolic compounds, commercially available	[186]
Cy	-	[189]
Cy3-glc (extracted and purified anthocyanin)	Tart cherries (*Prunus cerasus* fruits)	[188]
Cy3-glc	-	[187]
Mv	-	[81]

^1^ Established by LC-MS, ^2^ HPLC, ^3^ pH Differential Method.
